# 

**Published:** 1856-12

**Authors:** 


*To Correspondents.*—We are sadly in arrears in our correspondence, and positively have not the time to return answers to many letters which deserve a response. Communications from Drs. Maxson, Nichols, House, Crandall, and O. C. Gibbs, are on file for our next number; which will be one worthy to begin the new year, both in the variety and the ability of its contributions. This generous support from our friends, an increasing subscription list, and cheerful prospects in the future, start us forward in the New Year, 1857, with renewed energy and hopefulness.

---

*Dropsy following Scarlatina.*—From 1848 to 1852, there died in London, 11,566 with scarlatina. Of this number 1,575 were affected with dropsy succeeding that disease.—*British Review,* quoted in *Annuaire des Sciences Médicales.* 1856.

---

Our readers will notice that we have been compelled to omit our eclectic department, from the pressure of original matter. Many communications have also gone over to next month. We can get along, resignedly, with this sort of trouble. Book Publishers who have sent us books for reviews must "wait a little longer."

